# Stratifin accelerates progression of lung adenocarcinoma at an early stage

**DOI:** 10.1186/s12943-015-0414-1

**Published:** 2015-07-30

**Authors:** Aya Shiba-Ishii, Yunjung Kim, Toshihiro Shiozawa, Shinji Iyama, Kaishi Satomi, Junko Kano, Shingo Sakashita, Yukio Morishita, Masayuki Noguchi

**Affiliations:** Department of Pathology, Faculty of Medicine, University of Tsukuba, 1-1-1 Tennodai, Tsukuba-shi, Ibaraki 305-8575 Japan; Department of Pathology, Tokyo Medical University Ibaraki Medical Center, Inashiki-gun, Ibaraki Japan

**Keywords:** Stratifin, Tg-SPC-SFN^+/−^, Lung adenocarcinoma, Malignant progression, NNK

## Abstract

**Backgrounds:**

Adenocarcinoma in situ (AIS) of the lung has an extremely favorable prognosis. However, early but invasive adenocarcinoma (eIA) sometimes has a fatal outcome. We had previously compared the expression profiles of AIS with those of eIA showing lymph node metastasis or a fatal outcome, and found that stratifin (SFN, 14-3-3 sigma) was a differentially expressed gene related to cell proliferation. Here, we performed an *in vivo* study to clarify the role of SFN in initiation and progression of lung adenocarcinoma.

**Findings:**

Suppression of SFN expression in A549 (a human lung adenocarcinoma cell line) by siSFN significantly reduced cell proliferation activity and the S-phase subpopulation. *In vivo*, tumor development or metastasis to the lung was reduced in shSFN-transfected A549 cells. Moreover, we generated SFN-transgenic mice (Tg-SPC-SFN^+/−^) showing lung-specific expression of human SFN under the control of a tissue-specific enhancer, the SPC promoter. We found that Tg-SPC-SFN^+/−^ mice developed lung tumors at a significantly higher rate than control mice after administration of chemical carcinogen, NNK. Interestingly, several Tg-SPC-SFN^+/−^ mice developed tumors without NNK. These tumor cells showed high hSFN expression.

**Conclusion:**

These results suggest that SFN facilitates lung tumor development and progression. SFN appears to be a novel oncogene with potential as a therapeutic target.

**Electronic supplementary material:**

The online version of this article (doi:10.1186/s12943-015-0414-1) contains supplementary material, which is available to authorized users.

## Findings

Lung cancer is the most common cause of global cancer-related mortality, and the most common histological type is adenocarcinoma [1]. Noguchi *et al.* [2] have demonstrated that adenocarcinoma *in situ* (AIS) has an extremely favorable prognosis, with a 5-year survival rate of 100 %. AIS shows stepwise progression to early but invasive adenocarcinoma (eIA), which has a relatively poorer outcome, the 5-year survival rate being 75 % [2, 3]. We previously compared the gene expression profiles of AIS with those of eIA associated with lymph node metastasis or a fatal outcome, and screened the differentially expressed genes by cDNA microarray. Among genes showing significantly higher expression in eIA than in AIS, we finally focused on stratifin (SFN, 14-3-3 sigma) [4], and subsequently clarified that its overexpression in eIA is associated with demethylation of its promoter region [5].

14-3-3 is a highly conserved, ubiquitously expressed protein family, associated with many different cellular processes. Among the seven human 14-3-3 isoforms (beta, epsilon, eta, gamma, tau, zeta, and sigma), 14-3-3 sigma has been linked to cancer most directly. 14-3-3 sigma (SFN) was originally identified as a p53-inducible gene that is responsive to DNA-damaging agents [6, 7]. These findings define SFN as a negative regulator of cell cycle progression. However, our analysis of SFN expression in lung adenocarcinoma indicated that it is overexpressed in tumor cells to a greater extent than in normal lung epithelium, and that this overexpression stimulates tumor growth [4]. On the basis of these findings, we speculate that SFN in lung adenocarcinoma cells might have tissue-specific functions and regulate cell cycle progression in a positive manner.

**Fig. 1:**
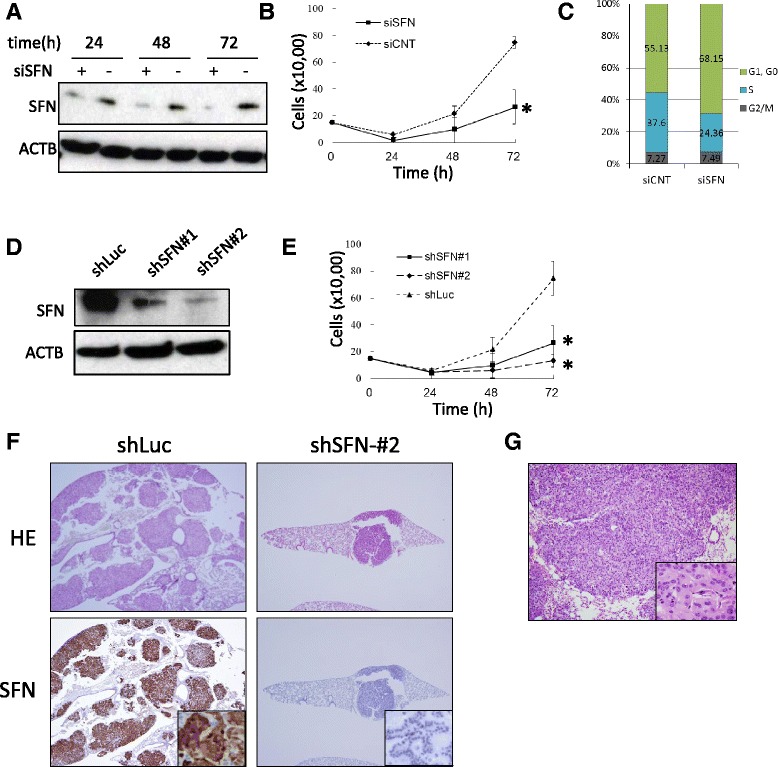
Functional analysis of SFN using A549 cells transfected with siSFN or shSFN. As a control, scrambled siRNA was used (siCNT). **a** SFN WB using A549 cells transfected with siSFN or siCNT. **b** Cell growth curve of siSFN-transfected A549. Cells were seeded in 24-well plates, cultured for the indicated time periods, and counted with a hematocytometer. Each group has five replicates. **c** Cell cycle analysis using siSFN-transfected A549. **d** Generation of stable SFN-knockdown A549 cell lines. Expression of SFN was significantly suppressed by stable expression of two independent shRNAs (#1 and #2) targeting SFN mRNA. shRNA for luciferase (shLuc) was used as a control. **e** Cell growth curve of A549 cells stably expressing shSFN. Each group has five replicates. **f** HE and SFN IHC were performed using FFPE specimens of the lungs collected from the mice injected with A549. Whereas numerous tumors showing SFN expression were observed in the mice intrabronchially injected with A549-shLuc, the mice intrabronchially injected with A549-shSFN#2 developed just one nodule each, measuring less than 1 mm. SFN expression was suppressed in the tumors that developed in the A549-shSFN#2 group. **g** HE image of tumors that developed in mice i.v. injected with A549-shLuc. Histologically, they are poorly differentiated adenocarcinoma. *: *p* >0.05

Here, we first examined the function of SFN in lung adenocarcinoma cells. All materials and methods are described in Additional file 1. Using the lung adenocarcinoma cell line A549 transfected with siSFN, cell proliferation, invasiveness, apoptosis, and senescence were analyzed respectively. Suppression of SFN led to a significant decrease in the number of cells and the S-phase subpopulation in comparison with controls (Fig. 1a-c, *p* <0.001, Student’s *t* test, Additional file 2: Figure S1). However, we were unable to detect any difference in invasiveness, apoptosis, or senescence between cells treated with siSFN and those treated with siCNT (Additional file 3: Figure S2a-d). Although SFN is reportedly related to apoptosis or senescence [8, 9], we were unable to detect any such association in the present study. This is likely because SFN does not bind to proteins such as BAX or BAD, which regulate apoptosis or senescence in lung adenocarcinoma (data not shown, personal communication). We are investigating the functional network of SFN in lung adenocarcinoma, and the results will be published in the near future.

We next induced stable knockdown of SFN using two individual shRNAs (shSFN#1 and shSFN#2). These shRNAs significantly inhibited the expression of SFN in A549 (Fig. 1d) and reduced the degree of cell growth (Fig. 1e). To evaluate the oncogenic activity of SFN, we injected A549-shSFN#1 and -shSFN#2 intrabronchially into SCID mice, and at 4 weeks after injection [10], all the mice were sacrificed and their lungs were collected. It was found that A549-shSFN#1 and A549-shSFN#2 formed significantly fewer tumors than the control cells (Table 1). Whereas the mice injected with A549-shLuc developed tumors up to 4 mm in size and the number of such tumors was high, the mice injected with A549-shSFN#2 developed just one tumor each, and each tumor was less than 1 mm in size (Fig. 1f). SFN expression was suppressed in the tumors that developed in the A549-shSFN#2 group relative to those that developed in the A549-shLuc group (Fig. 1f).

**Table 1:** Tumorigenicity of A549-shSFN in SCID mice

	shRNA	T (%)	D (%)	DM (%)
Intrabronchial	shSFN#1	0/4 (0)	0/4 (0)	0/4 (0)
(*n* = 4)	shSFN#2	2/4 (50)	0/4 (0)	0/4 (0)
	shLuc	4/4 (100)	1/4 (25)	0/4 (0)
Intravenous	shSFN#1	0/4 (0)	0/4 (0)	0/4 (0)
(*n* = 4)	shSFN#2	0/4 (0)	0/4 (0)	0/4 (0)
	shLuc	4/4 (100)	3/4 (75)	0/4 (0)

*T* tumor development, *D* intrathoracic dissemination by gross appearance, *DM* distant metastasis. Liver and brain were analyzed as examples of common sites of lung cancer metastasis

Moreover, in order to examine the effect of SFN on the metastatic potential of lung adenocarcinoma cells, we intravenously injected A549-shSFN#1 and -shSFN#2 into SCID mice, and sacrificed the mice 8 weeks after injection. We found that A549-shSFN#1 and A549-shSFN#2 did not form any tumors, whereas control cells formed numerous poorly differentiated adenocarcinomas in the lungs (Fig. 1g, Table 1). These data indicated that knockdown of SFN suppressed not only lung tumor formation but also the metastatic potential of lung adenocarcinoma.

While down-regulation of SFN *in vitro* had no effect on cancer cell migration and invasion (Additional file 3: Figure S2), suppression of SFN expression reduced the dissemination/metastasis of xenograft tumors (Table 1). Although the results of the *in vitro* and *in vivo* assays appear contradictory, we consider that growth reduction of A549-shSFN reduced the activity of the tumor cells in mice and finally affected their metastatic or invasive capacity.

**Fig. 2:**
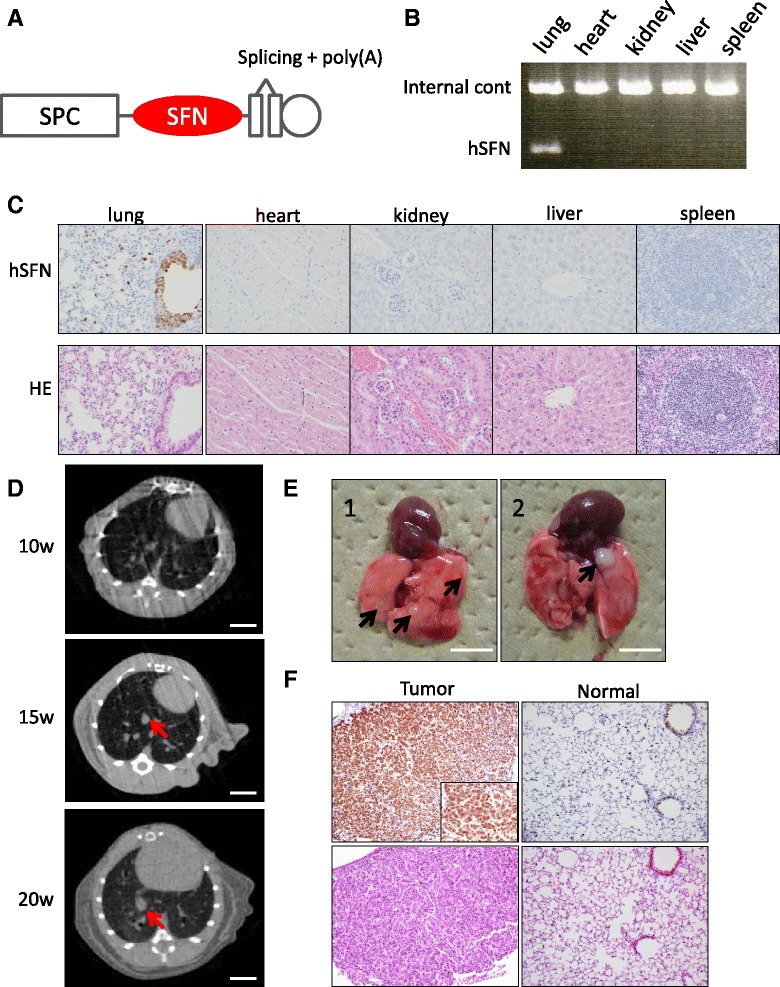
Generation of Tg-SPC-SFN^+/−^. **a** A cDNA for SFN was inserted between the SPC promoter and both the splicing and polyadenylation (polyA) signal sequences. **b** RT-PCR using cDNA extracted from lung, heart, kidney, liver, and spleen of representative Tg-SPC-SFN^+/−^ mice. **c** hSFN IHC using FFPE specimens of each of the organs from representative Tg-SPC-SFN^+/−^ mice. Transgene expression (hSFN) was detected only in lung. Magnification: ×200. **d** CT image of representative Tg-SPC-SFN^+/−^ lungs 10, 15, and 20 weeks after NNK administration. Arrows indicate suspected tumors. Scale bar: 10 mm. **e** Gross appearance of lungs of Tg-SPC-SFN^+/−^ mice administered NNK (1) and saline (2). Arrows indicates tumors. Scale bar: 5 mm. **f** hSFN IHC using FFPE specimens of tumors and adjacent normal lung from Tg-SPC-SFN^+/−^mice. Magnification: ×200

Next, to generate transgenic mice exhibiting lung-specific expression of human SFN (hSFN), we ligated a fragment of the surfactant protein C (SPC) promoter to a cDNA for hSFN (Fig. 2a), which was attached to a polyadenylation signal. The transgenic DNA was then injected into pronuclear-stage embryos of ICR (Institute of Cancer Research) mice, and the resulting offspring were screened by genotyping PCR for the presence of the transgene. Twelve founder mice positive for transgene integration were obtained. To confirm the lung-specific expression of the transgene, we performed RT-PCR and IHC to detect hSFN mRNA and protein in the F1 mice. The transgene was expressed in lung tissue (type II alveolar epithelial cells and basal cells in bronchial wall) but not in heart, kidney, liver, or spleen (Fig. 2b, c). We selected two lines that showed the highest hSFN expression in lung (lines 109 and 130, Additional file 4: Figure S3).

4-(Methylnitrosamino)-1-(3-pyridyl)-1-butanone (NNK), a tobacco-specific and highly potent pulmonary carcinogen, induces lung adenoma and adenocarcinoma in various species [11]. Our preliminary experiment showed that 18 % of WT ICR mice developed lung tumors 20 weeks after intraperitoneal (i.p.) administration of NNK. In order to observe the tumorigenic activity of SFN, Tg-SPC-SFN^+/−^ and WT ICR mice were i.p. administered 4 mg NNK, or saline as a control, and tumorigenicity was assessed for 20 weeks. Lungs of representative mice were periodically examined using animal CT. This revealed that some Tg-SPC-SFN^+/−^ developed pulmonary tumors from 15 weeks after NNK administration (Fig. 2d). At 20 weeks after NNK administration, 47.8 % of Tg-SPC-SFN^+/−^ (11/23) had developed lung tumors, whereas only 11.1 % of WT ICR (3/27) had done so (Table 2, Fig. 2e). This difference in the incidence of lung tumors was statistically significant (*p* <0.001, *χ*^2^ test). One Tg-SPC-SFN^+/−^ mouse developed three tumors, and another developed two simultaneously. The others developed one tumor each. Histologically, all of the tumors were poorly differentiated adenocarcinoma, but intrathoracic dissemination or metastasis was not observed until 20 weeks after NNK administration. Independent evaluation of male and female mice revealed no significant gender difference. Although it is possible that the transgene could have disrupted a locus leading to increased susceptibility to tumor formation, we consider that tumor formation in Tg-SPC-SFN^+/−^ was caused by hSFN overexpression because we also performed the same experiment using two transgenic lines independently, and the results were similar. All of the tumors that developed in Tg-SPC-SFN^+/−^ lung expressed hSFN abundantly (Fig. 2f). In contrast, tumors in WT ICR lung showed no hSFN expression, but slight mSFN expression (Additional file 5: Figure S4).

**Table 2:** Tumorigenicity of lung adenocarcinoma in Tg-SPC-SFN^+/−^ mice

Genotype	Wild type		Tg-SPC-SFN^+/−^	
i.p.	Saline	NNK	Saline	NNK
Nodule count				
0	7	24	5	12
1	0	3	1	10
2	0	0	1	0
3	0	0	0	1
Total	7	27	7	23
Proportion of mice with tumor(s)	0	11.1 %	28.6 %	47.8 %

Surprisingly, two of seven Tg-SPC-SFN^+/−^ mice (28.6 %) developed tumors even though they were not administered NNK (Table 2, Fig. 2e). These results indicate that SFN facilitates not only tumor progression but also tumor initiation, and that it works as an oncogene. Soda *et al.* found that 100 % of Tg-EML4-ALK mice developed hundreds of adenocarcinoma nodules in both lungs within a few weeks after birth [12]. Although the oncogenic activity of SFN is weaker than that of EML4-ALK fusion kinase, SFN also appears to have the potential to initiate lung adenocarcinoma.

A number of driver oncogenes have been reported, and many patients with lung adenocarcinoma are treated using various drugs targeting these oncogenes. Although these targeting drugs are initially effective, most cancers develop tolerance to them [13]. This phenomenon can be explained by complex alterations of oncogenes and anti-oncogenes in lung adenocarcinoma at the advanced stage [14]. However, according to the multistep carcinogenesis of lung adenocarcinoma [15], the number of such driver oncogenes in early-stage lung adenocarcinoma is expected to be limited, and SFN overexpression is one of the candidates suggested playing an important role in forced cell proliferation. During the course of slow but steady cell proliferation, tumor cells acquire several crucial gene alterations such as ALK translocation, p53 mutation and EGFR amplification. In the present study, we demonstrated that SFN is an oncogenic factor playing a role in the initiation and progression of early lung adenocarcinoma. Since alteration of SFN is observed in most lung adenocarcinomas, even AIS and minimally invasive adenocarcinoma, targeted therapy to block SFN might be a new treatment strategy for patients with early-stage lung adenocarcinoma. Although the mechanism underlying the function of SFN in lung adenocarcinoma progression has not been proved, further analysis of the molecular network of SFN in lung tumor cells is required.

## Additional files

Additional file 1:
**Materials and Methods.** (DOCX 20 kb) Additional file 2: Figure S1. Confirmation of the effect of specific siRNA for SFN. **A**) Relative SFN mRNA expression was analyzed by real-time RT-PCR. A549, A549 transfected with siSFN or siCNT. Although there was no difference in SFN expression between A549 and siCNT, SFN was strongly suppressed in siSFN. **B**) Relative SFN mRNA expression was analyzed 24, 48, and 72 h after siRNA transfection. siSFN continuously suppressed SFN expression for at least 72 h. (PDF 129 kb) Additional file 3: Figure S2. Functional analysis of SFN using A549 cells transfected with siSFN. **A**) Invasion assay was performed using A549 cells transfected with siSFN and incubated at 37 °C for 48 h. There was no significant difference between siSFN and siCNT. **B**) Scratch assay to assess migration [16]. A scratch wound was created by scraping the cell monolayer with a sterile 100-μL pipette, and the wounded cultures were then incubated at 37 °C for 16 h. Migrating cells in the wound area were photographed using a microscope and the size of the remaining wound was measured. Assays were performed three times using triplicate wells. **C**) Caspase-3/7 assay to assess apoptosis. A549 cells were transfected with siSFN and incubated at 37 °C for 48 h. UV-irradiated Jurkat cells as a positive control and unirradiated Jurkat cells as a negative control were also analyzed. **D**) Senescence-associated β-galactosidase (SA-β-Gal) assay to assess senescence. A549 cells were transfected with siSFN and incubated at 37 °C for 24 or 48 h. Quantification was performed by counting the positive cells present in 20 independent fields of view at × 10 magnification. *: *p* >0.05. (PDF 184 kb) Additional file 4: Figure S3. hSFN IHC using FFPE specimens of normal lung tissue from F1 Tg-SPC-SFN^+/−^ mice of 12 founder mice. Anti-hSFN antibody with no mouse cross-reactivity (Bethyl Laboratories, Montgomery, TX) was used as the first antibody. PC indicates positive control (A549 xenograft in nude mice), and NC indicates negative control (WT ICR mice). Lines 109 and 130 were selected for further analysis. Magnification: ×200. (PDF 392 kb) Additional file 5: Figure S4. SFN IHC using WT ICR-administered NNK. Anti-hSFN antibody with mouse cross-reactivity (IBL, Gumma, Japan) was used as the first antibody. Slight mSFN expression was detected only in tumor cells. (PDF 209 kb)

## Abbreviations

SFN: Stratifin
SCID: Severe combined immunodeficiency
ICR: Institute of Cancer Research
NNK: 4-(Methylnitrosamino)-1-(3-pyridyl)-1-butanone
SPC: Surfactant protein C
Tg: Transgenic mice
WT: Wild type
eIA: early invasive adenocarcinoma
AIS: Adenocarcinoma in situ
IHC: Immunohistochemistry
