# Supplementary material for: Stratifin accelerates progression of lung adenocarcinoma at an early stage
Source: Mol Cancer. 2015 Jul 30;14:142. doi: 10.1186/s12943-015-0414-1 (PMC4518688; doi:10.1186/s12943-015-0414-1)
Supplement: Additional file 1: — Materials and Methods. (DOCX 20 kb) [file 12943_2015_414_MOESM1_ESM.docx]

**Supplementary information**

Materials and Methods

**siRNA and shRNA**

SFN-specific siRNA (forward, 5'-ACCACGUUCUUAUAGGCUACUGAGA-3';　reverse, 5'-UCUCAGUAGCCUAUAAGAACGUGGU-3'; Life Technologies Corporation) was transfected into cells using a nucleic acid transfer agent, lipofectamine RNAiMAX (Life Technologies Corporation). As a control, Stealth RNAi™ siRNA Negative Control, Med GC (Life Technologies Corporation) was used.

SFN-specific shRNA#1 (GATCCCCTCTCAGTAGCCTATAAGAA TTCAAGAGATTCTTATAGGCTACTGAGATTTTTC) and shRNA#2 (GATCCCC GAGCCAGTCTGATCCAGAATTCAAGAGATTCTGGATCAGACTGGCTCTTTTTC) ligated in a pSUPER RNAi system (OligoEngine) were used for stable knockdown of SFN. The ligation was performed in accordance with the manufacturer’s instructions. For selection of stable SFN-knockdown clones by transfected shRNA vectors, A549 cells were cultured in the presence of 1.5 µg/mL puromycin (Sigma). As a control, luciferase-specifc shRNA was used. Generation and transfection of expression vectors have been described elsewhere [16].

**Cell proliferation analysis**

Cells were seeded into 24-well plates, cultured for the indicated time periods, and counted with a hemacytometer. Each group had five replicates. Cell cycle analysis was carried out using a FITC BrdU flow kit (BD, Franklin Lakes, NJ) and a FACScalibur (BD).

**Transplantation of tumor cells**

Animal experiments were carried out humanely in accordance with the Regulations for Animal Experiments of the University of Tsukuba and Fundamental Guidelines for Proper Conduct of Animal Experiments and Related Activities in Academic Research Institutions under the jurisdiction of the Ministry of Education, Culture, Sports, Science, and Technology of Japan and with approval from the Institutional Animal Experiment Committee of our university.

1.5 × 10^6^ A549 cells that were stably transfected with shSFN in 0.1 mL PBS were injected via the tail vein into 6-week-old SCID mice (CLEA Japan). At 8 weeks after injection, the mice were sacrificed. Also, 1.0 × 10^7^ A549 cells that were stably transfected with shSFN in 0.05 mL PBS were injected into the peripheral bronchus of 6-week-old SCID mice (CLEA Japan). Because it has been reported that the majority of mice die within 4 weeks after intrabronchial transplantation [10], the mice were sacrificed at 4 weeks after injection,

**Generation of transgenic mice**

Human SFN (hSFN) fragment was amplified using the following primers from a SFN expression vector constructed elsewhere [4]: forward, CGCGGCCGCATGGAGAGAGCCAGTCTGAT, reverse, CGCGGCCGCGTTTAAACCTAGCTCTGGGGCTCCT. A cDNA fragment encoding hSFN was ligated to the SPC promoter as well as to the splicing and polyadenylation signals. The resulting expression cassette was injected into the pronuclei of fertilized oocytes derived from ICR mice. The injected embryos were then transferred into the oviduct of pseudopregnant ICR females. ICR mice were purchased from Charles River Laboratories Japan (Kanagawa, Japan). These steps were performed at the Laboratory Animal Resource Center, University of Tsukuba (Ibaraki, Japan). The presence of the transgene was examined by genotyping PCR using DNA from the tail of founder mice.

For detection of mRNAs and proteins from the transgene, RT-PCR and IHC of hSFN were carried out as described previously [4]. The antibody against hSFN was purchased from GeneTex, Inc. (San Antonio, TX).

**Chemical carcinogenesis experiment**

Generated Tg-SPC-SFN+/- and WT ICR mice were given a single i.p. dose of 4 mg NNK (Toronto Research Chemicals, Toronto, Canada) in saline. Sequential examination of the lungs was performed with an X-ray CT apparatus for experimental animals (LCT-100, Hitachi Aloka Medical, Tokyo, Japan) at 10, 15, and 20 weeks after the carcinogen treatment. All the mice were sacrificed at 20 weeks after the treatment. The lungs were then immediately excised and fixed in formalin.

**Western blotting**

Total cell lysates were prepared on ice with M-PER^TM^ Mammalian Protein Extraction Reagent (Pierce, Rockford, IL) containing a protease inhibitor cocktail (Sigma) and phosphatase inhibitor cocktail (Sigma). The lysates were centrifuged for 10 min at 4°C, and the insoluble fraction was discarded. The total protein in the soluble lysates was measured using a BCA protein assay kit (Pierce). Total protein aliquots (20 μg) were mixed with sample buffer, denatured at 95°C for 5 min, and electrophoresed on 10% Tris-HCl gel (Bio-Rad Laboratories, Hercules, CA). Proteins were transferred to polyvinylidene difluoride membranes using an iBlot^TM^ gel transfer system (Life Technologies Corporation). The blots were blocked and probed with the various antibodies, as indicated in the figure legends. After extensive washing, immunoreactivity was detected with specific secondary antibodies conjugated to horseradish peroxidase. Protein bands were visualized using SuperSignal West Femto® extended duration substrate (Pierce) and X-ray film (BioMax XAR film; Kodak, Rochester, NY). Antibodies used for Western blotting were obtained from the following companies: SFN, Immuno-Biological Laboratories Co., Ltd. (Gunma, Japan); β-actin, Sigma-Aldrich (St Louis, MO).

**Invasion assay**

Cell invasion potential was assessed using a cell invasion assay kit (Millipore) in accordance with the manufacturer’s protocol. The kit had a system for evaluating the invasion of tumor cells through a basement membrane model. A549 cells were transfected with siSFN and incubated at 37°C for 48 h.

**Scratch assay**

A scratch wound was created by scraping the cell monolayer with a sterile 100-μL pipette, as described previously [15]. We changed the culture medium immediately after scraping to prevent the medium from being conditioned with cell debris and factors released from the detached cells. The wounded cultures were then incubated in DMEM/F-12 and 10% FBS for 16 h until the following studies were conducted. At the indicated time, migrating cells in the wound area were photographed using a microscope and the size of the remaining wound was measured. Assays were performed three times using triplicate wells.

**Apoptosis detection**

Caspase 3/7 was measured as an apoptosis indicator using Caspase-Glo 3/7 Assay (Promega, Tokyo, Japan) in accordance with the manufacturer’s instructions. A549 cells were transfected with siSFN and incubated at 37°C for 48 h. UV-irradiated Jurkat cells as a positive control and unirradiated Jurkat cells as a negative control were also analyzed.

**Senescence detection**

Cell senescence was assessed by detecting senescence-associated β-galactosidase activity at pH 6.0 (SA-β-Gal) using a Senescence β-Galactosidase Staining Kit (Cell Signaling Technology) in accordance with the manufacturer’s instructions. A549 cells were transfected with siSFN and incubated at 37°C for 24 or 48 h. Quantification was performed by counting the positive cells present in 20 independent fields of view at ×10 magnification.
