# Supplementary figures and images for: Stratifin accelerates progression of lung adenocarcinoma at an early stage
Source: Mol Cancer. 2015 Jul 30;14:142. doi: 10.1186/s12943-015-0414-1 (PMC4518688; doi:10.1186/s12943-015-0414-1)

Additional Fig. 1

**A**

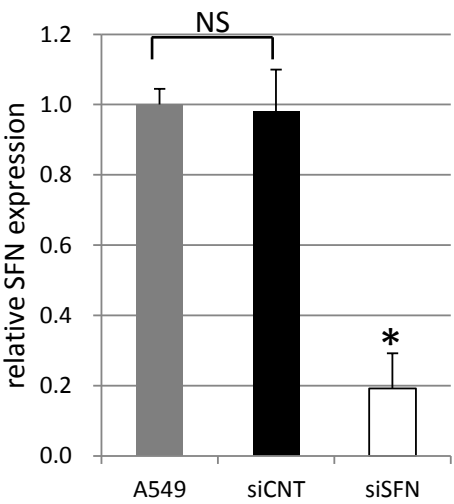

**B**

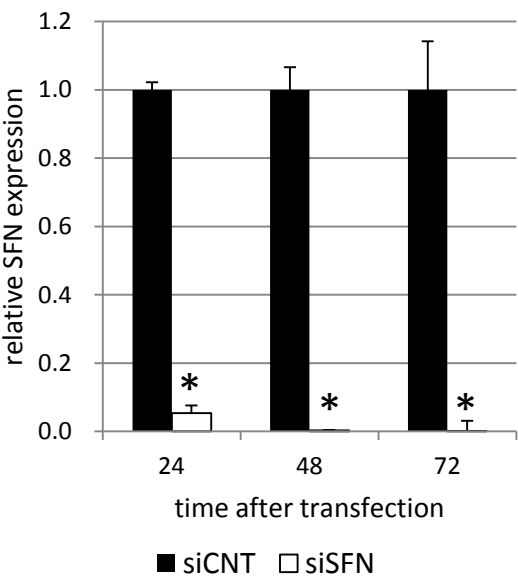

Supplement: Additional file 2: Figure S1. — Confirmation of the effect of specific siRNA for SFN. A) Relative SFN mRNA expression was analyzed by real-time RT-PCR. A549, A549 transfected with siSFN or siCNT. Although there was no difference in SFN expression between A549 and siCNT, SFN was strongly suppressed in siSFN. B) Relative SFN mRNA expression was analyzed 24, 48, and 72 h after siRNA transfection. siSFN continuously suppressed SFN expression for at least 72 h. (PDF 129 kb) [file 12943_2015_414_MOESM2_ESM.pdf]

Additional Fig. 2

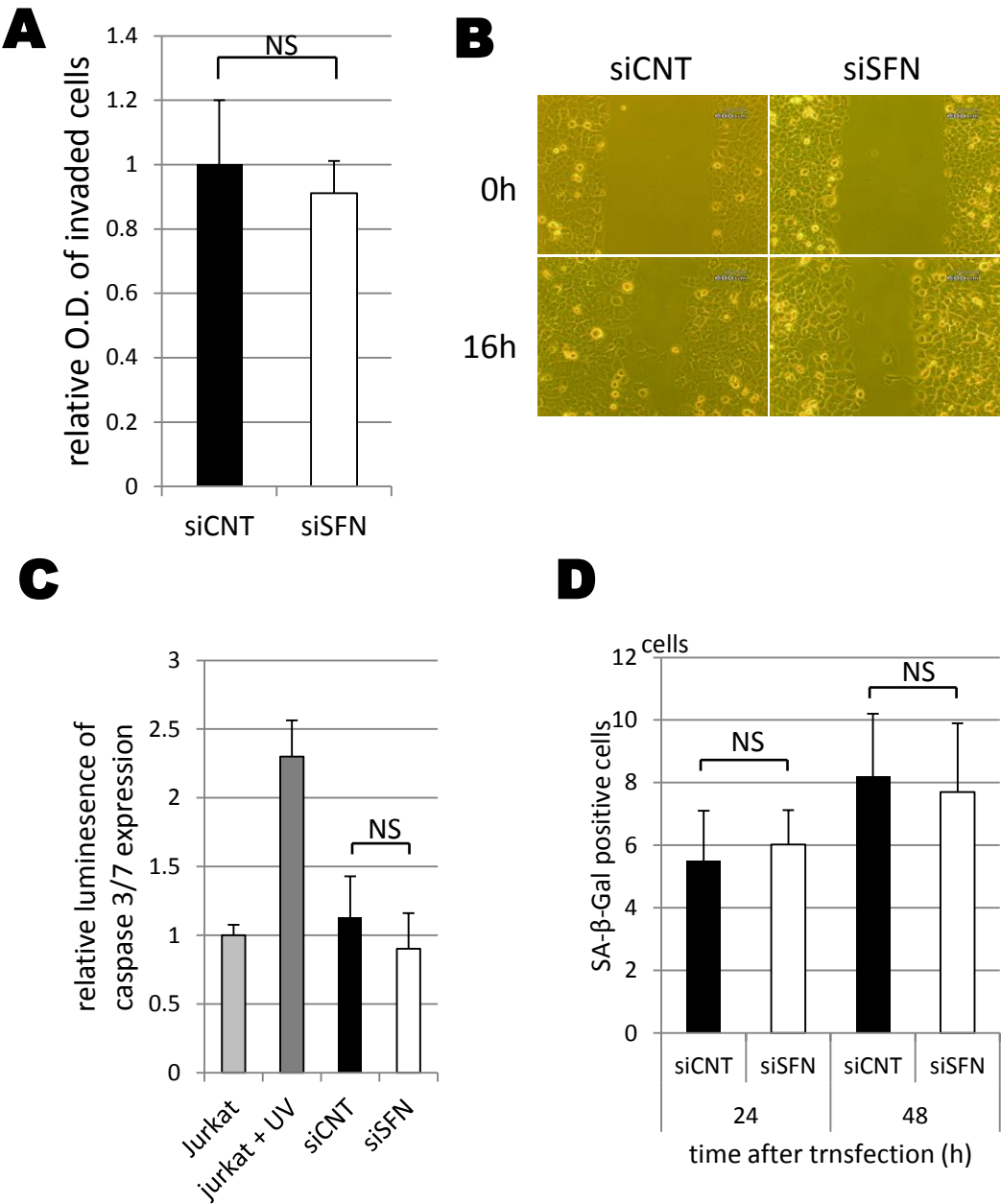

Supplement: Additional file 3: Figure S2. — Functional analysis of SFN using A549 cells transfected with siSFN. A) Invasion assay was performed using A549 cells transfected with siSFN and incubated at 37 °C for 48 h. There was no significant difference between siSFN and siCNT. B) Scratch assay to assess migration [16]. A scratch wound was created by scraping the cell monolayer with a sterile 100-μL pipette, and the wounded cultures were then incubated at 37 °C for 16 h. Migrating cells in the wound area were photographed using a microscope and the size of the remaining wound was measured. Assays were performed three times using triplicate wells. C) Caspase-3/7 assay to assess apoptosis. A549 cells were transfected with siSFN and incubated at 37 °C for 48 h. UV-irradiated Jurkat cells as a positive control and unirradiated Jurkat cells as a negative control were also analyzed. D) Senescence-associated β-galactosidase (SA-β-Gal) assay to assess senescence. A549 cells were transfected with siSFN and incubated at 37 °C for 24 or 48 h. Quantification was performed by counting the positive cells present in 20 independent fields of view at × 10 magnification. *: p >0.05. (PDF 184 kb) [file 12943_2015_414_MOESM3_ESM.pdf]

Additional Fig. 3

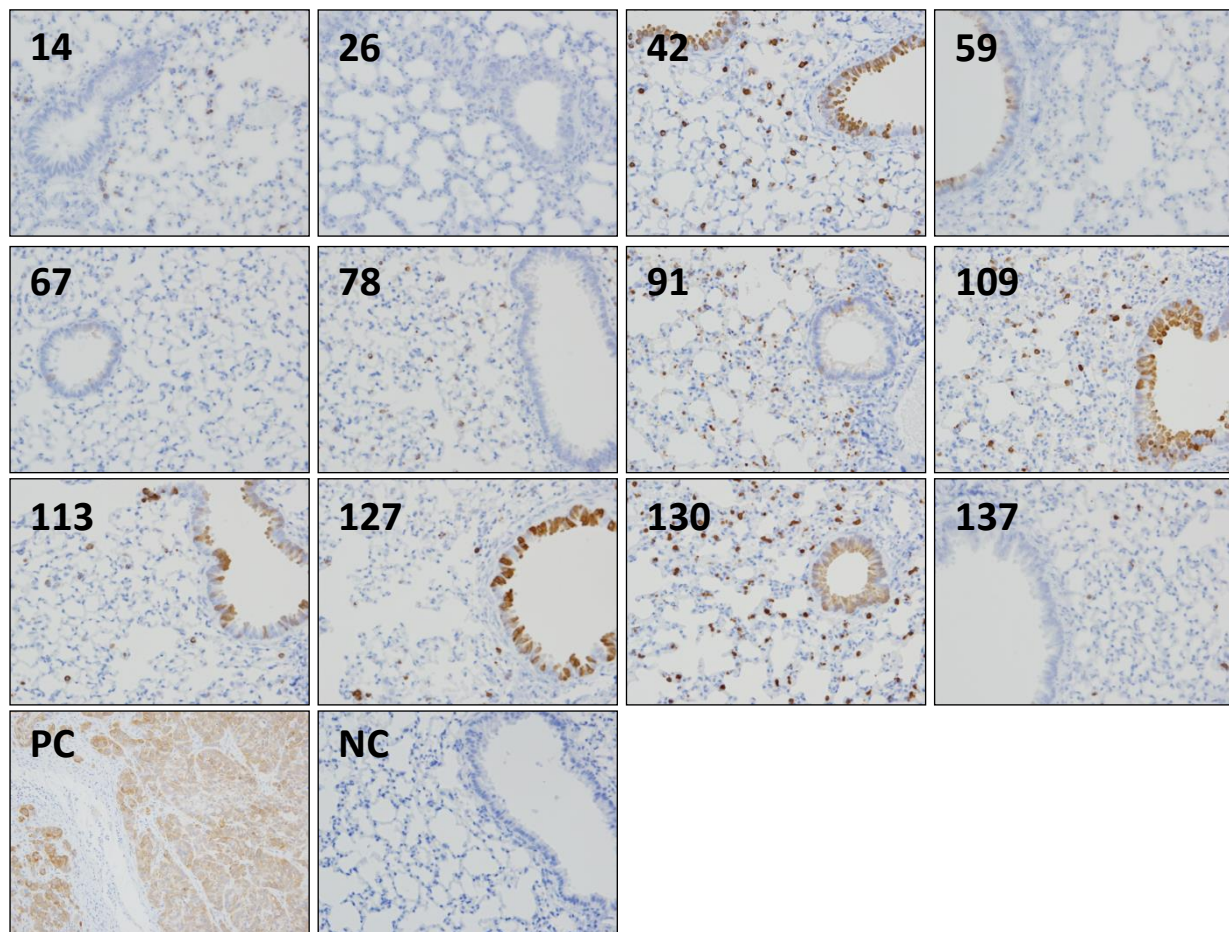

Supplement: Additional file 4: Figure S3. — hSFN IHC using FFPE specimens of normal lung tissue from F1 Tg-SPC-SFN+/− mice of 12 founder mice. Anti-hSFN antibody with no mouse cross-reactivity (Bethyl Laboratories, Montgomery, TX) was used as the first antibody. PC indicates positive control (A549 xenograft in nude mice), and NC indicates negative control (WT ICR mice). Lines 109 and 130 were selected for further analysis. Magnification: ×200. (PDF 392 kb) [file 12943_2015_414_MOESM4_ESM.pdf]

Additional Fig. 4

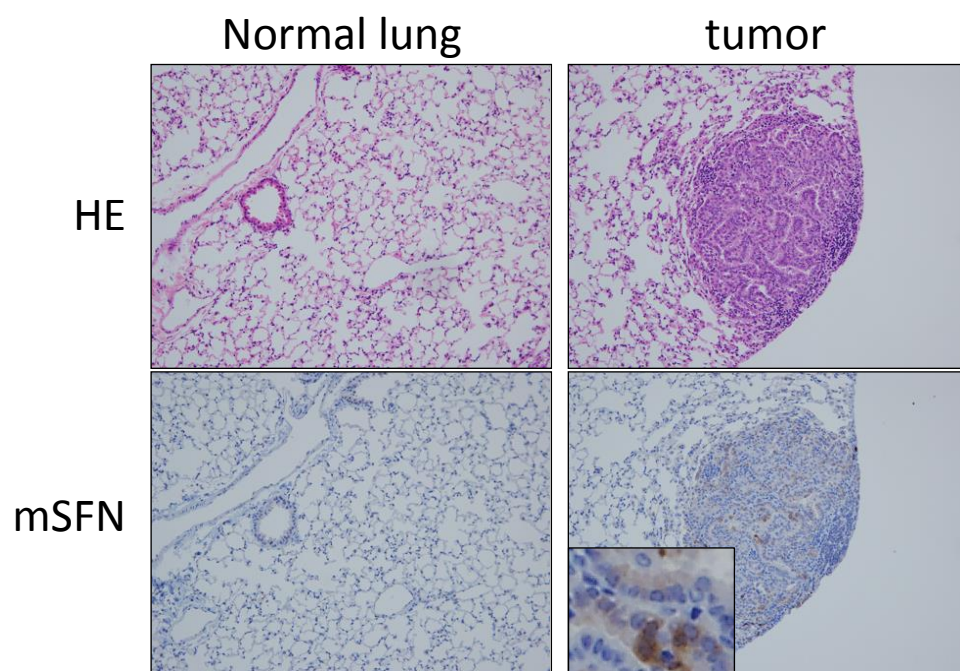

Supplement: Additional file 5: Figure S4. — SFN IHC using WT ICR-administered NNK. Anti-hSFN antibody with mouse cross-reactivity (IBL, Gumma, Japan) was used as the first antibody. Slight mSFN expression was detected only in tumor cells. (PDF 209 kb) [file 12943_2015_414_MOESM5_ESM.pdf]
